# Effect of Calcium Chloride Hydrothermal Treatment of Titanium on Protein, Cellular, and Bacterial Adhesion Properties

**DOI:** 10.3390/jcm9082627

**Published:** 2020-08-13

**Authors:** Takuya Haraguchi, Yasunori Ayukawa, Yukie Shibata, Toru Takeshita, Ikiru Atsuta, Yoichiro Ogino, Noriyuki Yasunami, Yoshihisa Yamashita, Kiyoshi Koyano

**Affiliations:** 1 Section of Implant and Rehabilitative Dentistry, Division of Oral Rehabilitation, Faculty of Dental Science, Kyushu University, 3-1-1 Maidashi, Higashi-ku, Fukuoka 812-8582, Japan; haraguchi0711@dent.kyushu-u.ac.jp (T.H.); n.yasu@dent.kyushu-u.ac.jp (N.Y.); koyano@dent.kyushu-u.ac.jp (K.K.); 2 Section of Preventive and Public Health Dentistry, Division of Oral Health, Growth and Development, Faculty of Dental Science, Kyushu University, 3-1-1 Maidashi, Higashi-ku, Fukuoka 812-8582, Japan; yukie@dent.kyushu-u.ac.jp (Y.S.); taketooo@dent.kyushu-u.ac.jp (T.T.); yoshi@dent.kyushu-u.ac.jp (Y.Y.); 3 Division of Advanced Dental Devices and Therapeutics, Faculty of Dental Science, Kyushu University, 3-1-1 Maidashi, Higashi-ku, Fukuoka 812-8582, Japan; atyuta@dent.kyushu-u.ac.jp; 4 Section of Fixed Prosthodontics, Division of Oral Rehabilitation, Faculty of Dental Science, Kyushu University, 3-1-1 Maidashi, Higashi-ku, Fukuoka 812-8582, Japan; ogino@dent.kyushu-u.ac.jp

**Keywords:** calcium-hydrothermal treatment, titanium, osteoblast, epithelial cell, bacterial adhesion

## Abstract

Topographical modification of the dental implant surface is one of the main topics for the improvement of the material, however, the roughened surface has some risks for peri-implantitis. A hydrothermal treatment (HT) of titanium with calcium chloride solution was reported to improve osseointegration and soft tissue sealing without changing the surface topography; however, its mechanism is unclear. We herewith investigated the interaction between extracellular matrix (ECM) protein and HT titanium. Furthermore, we also clarified the bacterial interaction. We employed two kinds of HT, HT with water (DW-HT) and HT with calcium chloride solution (Ca-HT). As a result, the adsorptions of both laminin-332 and osteopontin onto the Ca-HT surface were enhanced. In contrast, the adsorption of albumin, which was reported to have no cell adhesion capacity, was not influenced by Ca-HT. Osteoblast adhesion onto Ca-HT was also enhanced. Although Ca-HT was reported to enhance both epithelial cell attachment strength and in vivo peri-implant epithelial bonding, the number of epithelial cell attachment was not increased even after HT. Ca-HT had no impact in the adhesion of *Streptococcus gordonii*. These results suggest that Ca-HT enhances cell adhesion onto titanium without increasing bacterial adhesion, and the improvement of ECM protein adsorption is supposed to contribute to cell adhesion.

## 1. Introduction

Accumulated evidence has proven the long-term sustainability of dental implants as a treatment modality for edentulous patients [1,2]. To improve the success rate of implants, continuous efforts have been made to develop better quality materials. The evolution of implant materials has mainly focused on topographical alterations of the surface to enhance rapid osseointegration. However, although a rough implant surface may promote rapid osseointegration, it may also lead to peri-implantitis [3]. Peri-implantitis is induced by peri-implant plaque accumulation [4], which may result in tissue inflammation and bone resorption [5]. Although one study reported the incidence of peri-implantitis to be 22% [6], a gold standard for its treatment has not yet been established, and peri-implantitis is still one of the most common reasons for implant failure [7]. Clearly, the development of an implant surface with a preventive effect against peri-implantitis is a significant goal.

Hemidesmosomes (HDs) are known as cell adhesion structures that facilitate the adhesion between the enamel of the tooth and junctional epithelial cells. HDs are also formed between titanium and peri-implant epithelial cells; however, the bond between titanium and peri-implant soft tissue is reported to be much weaker than the bond between tooth and epithelial cells [8,9]. This is thought to be one reason for peri-implant tissue breakdown.

In 2017, the new classification of periodontal disease was established [10] and, in this classification, peri-implant conditions were classified into four statuses; that is, peri-implant health, peri-implant mucositis, peri-implantitis, and hard and soft tissue implant site deficiencies [11]. In this report, peri-implant mucositis can be characterized by an increase in probing depth owing to a decrease in probing resistance [11]. There is a possibility that the weakness of the connection between implant and peri-implant soft tissue induces the decrease in the resistance and subsequent pocket deepening.

Previous studies have suggested that hydrothermal treatment (HT) with calcium chloride solution (CaCl_2_) enhances bone–titanium implant contact [12]. In addition, this treatment has also been shown to enhance the attachment of gingival epithelial-like cells and fibroblast onto titanium disks [13,14,15,16,17]. An in vivo rodent study also revealed that this treatment enhanced peri-implant soft tissue bonding [18]. In addition, because this treatment exerts almost no impact on the surface topography [13,15,18], it seems to be advantageous for increasing the functionality of the implant surface. However, the detailed mechanism of the effects of HT with CaCl_2_ (Ca-HT) on osteoblastic and epithelial cell attachment remain unclear. Given that cell adhesion onto the substrata is established by interaction with integrin or other cell membrane protein–extracellular matrix (ECM) proteins [19], it can be speculated that the surface of Ca-HT titanium provides superior adsorption of the cell adhesion-related ECM protein. However, the possible superior protein adsorption capability of Ca-HT titanium also poses some risk of increasing bacterial adhesion onto the surface.

The aim of this study was to investigate the mechanism of the effect of Ca-HT on epithelial and osteoblastic cell adhesion by studying the adsorption of laminin-332, osteopontin, and albumin, and to explore the initial adhesion characteristics of bacteria onto Ca-HT titanium.

## 2. Materials and Methods

### 2.1. Titanium Plates

Commercially available pure titanium plates (diameter 5 mm, thickness 1.5 mm; ASTM B348-GR2) were used in the present study. These were divided into three groups. The non-process (NP) group specimens received no treatment. The HT with water (DW-HT) group received hydrothermal treatment with distilled water at 200 °C for 24 h. The Ca-HT group received hydrothermal treatment with a 10 mmol/L solution of CaCl_2_ at 200 °C for 24 h, as reported previously [13]. The contact angles of a 1 μL H_2_O droplet were measured using a contact angle meter (Drop Master, Kyowa Interface Science, Niiza, Japan). All experiments were started immediately after the completion of the hydrothermal treatment.

### 2.2. Protein Adsorption Assay

Pure titanium plates were treated as above. The center line average roughness (Ra) was measured using 3D laser microscopy (VK-9710, Keyence, Osaka, Japan).

The titanium plates were coated with recombinant human osteopontin (rhOPN; 1433-OP-050, R & D Systems, Minneapolis, MN, USA) or recombinant human laminin-332 (rhLN; ReproCELL, Yokohama, Japan). rhOPN and rhLN were diluted with 0.01 M phosphate-buffered saline (PBS) at concentrations of 2.5 μg/mL and 0.5 μg/mL, respectively, and titanium plates were immersed in one of these solutions for 1 h. After rinsing off the unbound protein with PBS, the titanium plates were treated with 10% normal rabbit serum or normal goat serum (Nichirei Bioscience, Tokyo, Japan) for 10 min to prevent nonspecific adsorption of antibodies, and then the titanium plates were incubated with 5 μg/mL diluted goat anti-human OPN polyclonal antibody (AF1433-SP, R & D Systems, Minneapolis, MN, USA) or 4 μg/mL diluted mouse anti-human laminin-332 monoclonal antibody (P3H9-2, Santa Cruz Biotechnology, Dallas, TX, USA) for 1 h. After rinsing unbound antibodies off with PBS, the plates were treated with 10 μg/mL of fluorescein-conjugated rabbit anti-goat IgG or goat anti-mouse IgG (Invitrogen, Carlsbad, CA, USA) for 30 min. After the reaction, the titanium plates were rinsed with distilled water, and fluorescent intensity was measured using a multimode microplate reader (Infinite F200 Pro, Tecan, Salzburg, Austria). Fluorescent intensity was measured at 16 points on each plate and the mean value was calculated. All reactions were conducted at room temperature. The measurement was conducted five times using five separate plates in each group. To establish a baseline fluorescent intensity value, the procedures were performed without primary antibodies on three kinds of titanium plates (NP, DW-HT, Ca-HT), and fluorescent intensity values of the respective controls were subtracted from those of the experimental groups. All fluorescent intensity values were divided by the average value of NP. Thus, the intensity values are expressed as a ratio to NP.

The titanium plates were also coated with bovine serum albumin (BSA, Thermo Scientific, Rockford, IL, USA). After 1 h of incubation into 1 mg/mL BSA solution, unbound protein was rinsed with PBS. Then, BSA bounded onto plates was measured using Pierce^TM^ BCA Protein Assay Kit (Thermo Scientific, Rockford, IL, USA) with a spectrophotometer (NJ-2300, Biotec, Tokyo, Japan), according to the manufacturer’s instruction. Absorbance values were compared as above.

### 2.3. Cell Culture

To elucidate the osteoblastic or epithelial cell attachment characteristics toward the treated titanium plates, a cell culture experiment was performed.

The GE1 mouse gingival epithelial cell line was provided by RIKEN BRC (Tsukuba, Japan) through the National Bio-Resource Project of the Ministry of Education, Culture, Sports, Science, and Technology, Japan. GE1 cells were cultivated in SFM-101 (Nissui Pharmaceutical, Tokyo, Japan) containing 1% fetal bovine serum (FBS) supplemented with 10 ng/mL mouse epidermal growth factor at 33 °C in a humidified atmosphere of 5% CO_2_ in air for 24 h [20]. The titanium plates (diameter 15 mm, thickness 1 mm) were pre-treated with 3% FBS for 8 h, and cells were seeded in a 0.5 mL volume onto each titanium plate at a density of 0.5 × 10^5^ cells per titanium plate in a 24-well plate (Falcon Labware, Oxford, UK). The MC3T3-E1 osteoblastic cells (RIKEN BRC) were cultured in alpha-minimum essential medium (Gibco, Grand Island, NY, USA) containing 10% FBS at 37 °C in a humidified atmosphere of 5% CO_2_ in air for 1 h.

### 2.4. Cell Staining

After cultivation, the cells were fixed with 4% paraformaldehyde for 10 min, washed with PBS, and then stained with actin using tetramethyl-rhodamine isothiocyanate-conjugated phalloidin (1:200 dilution; Sigma–Aldrich, St Louis, MO, USA). The cells were then mounted using mounting medium containing 6-diamidino-2-phenylindole (DAPI) (VECTASHIELD, Vector Laboratories, Burlingame, CA, USA) for nuclear staining. Cells were observed under a fluorescence microscopy (BZ-9000, Keyence, Osaka, Japan).

### 2.5. Attached Cell Count

The number of DAPI-positive nuclei was counted from 10 randomly-selected views (1455 μm × 1098 μm = 1.60 × 10^6^ μm^2^ per view) using software bundled with fluorescence microscopy [21,22]. The average number of DAPI-positive nuclei per one view was defined as the attached cell count.

### 2.6. Bacterial Culture

The strain used in the present study was *Streptococcus gordonii* ATCC 10558 and were grown in brain heart infusion (BHI; Difco, Grand Island, NY, USA) at 37 °C in 5% CO_2_. Unstimulated whole saliva was collected from four healthy adult volunteers, as reported previously [23]. Saliva was pooled and centrifuged at 12,000 *g* for 20 min at 4 °C. The resulting supernatant was sterilized through a 0.45 µm Minisart-plus filter (Sartorius Stedim Biotech, Göettingen, Germany). Titanium plates were placed into a 48-well microplate and soaked with 200 µL of saliva for 30 min at 37 °C. After washing with PBS, the microplate was inoculated with 20 µL bacteria-containing BHI (OD_550_ = 0.80 ± 0.05) and 200 µL of BHI supplemented with 1% (wt/vol) glucose. After incubation for 24 h at 37 °C, the liquid medium and unattached bacterial cells were removed, and loosely bound bacterial cells on the titanium plates were removed by washing with PBS. Adherent bacteria were then collected using ultrasonic vibration. The bacterial specimens were suspended in 300 µL lysis buffer (10 mM Tris-HCl containing 1% sodium dodecyl sulfate and 1 mM ethylenediaminetetraacetic acid), kept in ice during delivery to the laboratory, and then frozen at −30 °C. DNA was extracted as described previously [24,25]. To estimate bacterial counts, quantitative real-time PCR analysis was performed using a QuantiFast SYBR Green PCR kit (Qiagen, Hilden, Germany) in a StepOne Real-Time PCR System (Applied Biosystems, Foster City, CA, USA) according to the manufacturer’s instructions. The universal primers 806F (5′-TTA GAT ACC CYG GTA GTC C-3′) and 926R (5′-CCG TCA ATT YCT TTG AGT TT-3′) were used [26]. The 25 µL reaction mixture consisted of 1 µL template DNA, 0.1 µL of each primer (100 pmol/µL; final concentration, 400 nM), 12.5 µL Fast SYBR Green Master Mix (2×), and 11.3 µL sterile distilled water. The cycling conditions were 95 °C for 10 min, followed by 40 cycles of 95 °C for 3 s, and 60 °C for 30 s. Melting curves for the 16S rRNA amplicons were assessed for artifacts or nonspecific PCR products. The relative amounts of total bacteria were determined using the ∆∆Ct method, and DNA extracted from *Porphyromonas gingivalis* was used as a control.

### 2.7. Statistical Procedure

One-way analysis of variance (ANOVA) with Scheffe’s post hoc test was used for multiple comparison, using statistical software (Ekuseru-Toukei 2008, Social Survey Research Information, Tokyo, Japan). Values of *p* < 0.05 were considered to be statistically significant.

## 3. Results

### 3.1. The Surface Characteristics of Titanium Plates

The water contact angles of tested titanium plates were 86.2° (NP), 1.22° (DW-HT), and 1.87° (Ca-HT), the latter two of which represented superhydrophilicity.

The average Ra values of the five plates in each group were 0.332 μm (NP), 0.333 μm (DW-HT), and 0.348 μm (Ca-HT), and there were no statistically significant differences among the three groups.

### 3.2. Protein Adsorption Assay

**Figure 1:**
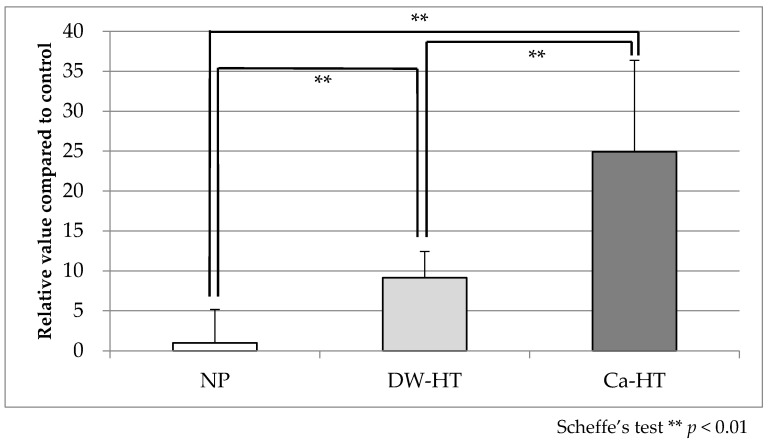
Recombinant human laminin-332 (rhLN) adsorption. There were statistically significant differences among the groups (*p* < 0.01). NP, non-process; DW-HT, hydrothermal treatment with water; Ca-HT, hydrothermal treatment with calcium chloride solution.

Adsorption of rhLN onto the substrata was 1.000 ± 4.157 (NP), 9.144 ± 3.282 (DW-HT), and 24.909 ± 11.476 (Ca-HT). Statistically significant differences were observed among all three groups (Figure 1).

**Figure 2:**
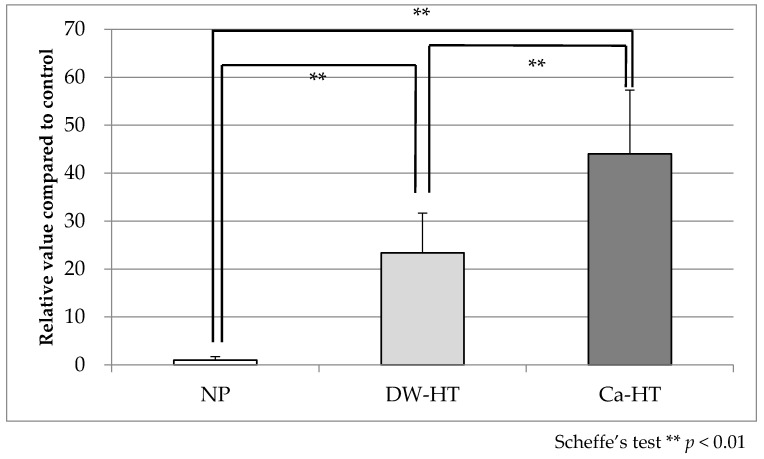
Recombinant human osteopontin (rhOPN) adsorption. There were statistically significant differences among the groups (*p* < 0.01).

Adsorption of rhOPN onto the substrata was 1.000 ± 0.728 (NP), 23.375 ± 8.25 (DW-HT) and 44.024 ± 13.268 (Ca-HT). Statistically significant differences were observed among all three groups (Figure 2).

**Figure 3:**
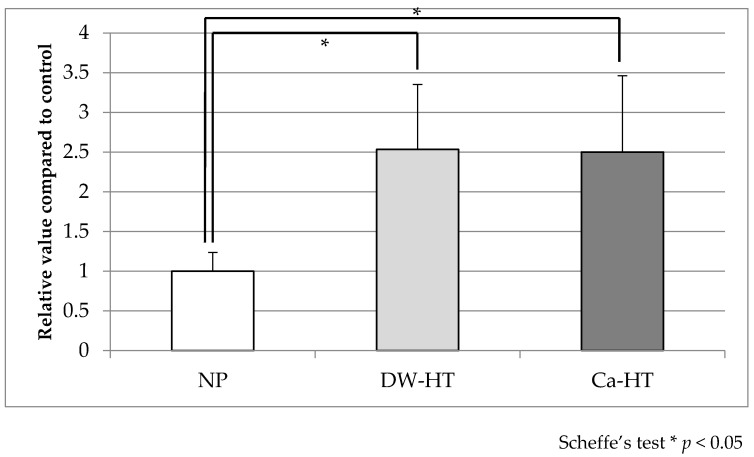
Bovine serum albumin (BSA) adsorption. BSA adsorption was significantly higher in both HT groups than in the NP group. No statistical differences were observed between the HT groups (*p* < 0.05).

BSA adsorption onto the substrata was 1.000 ± 0.264 (NP), 2.533 ± 0.916 (DW-HT), and 2.50 ± 1.074 (Ca-HT). Statistically significant differences were observed between NP and both HT groups; however, no statistically significant differences were observed between the DW-HT and Ca-HT groups (Figure 3).

### 3.3. Initial Cell Adhesion

**Figure 4:**
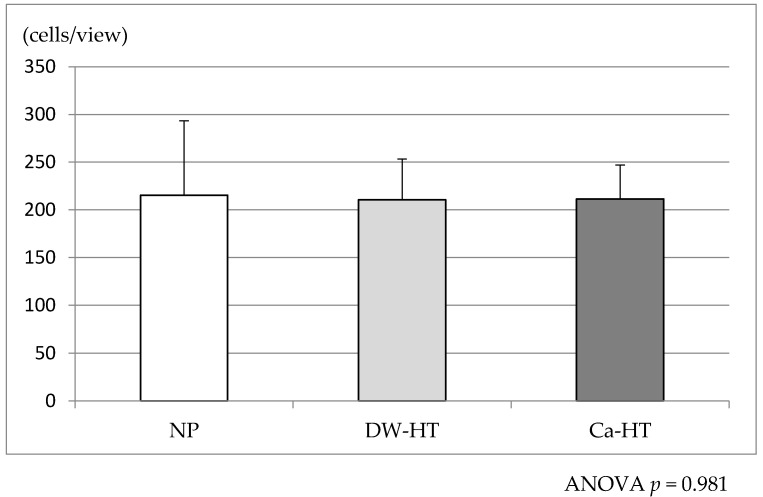
Adhesion of GE1 oral epithelial cells onto the substrata after 12 h of incubation. There were no significant differences in the attached cell count among the three groups. ANOVA, analysis of variance.

After 12 h of incubation of GE1 oral epithelial cells with the substrata, there were no significant differences in attached cell count among three groups (Figure 4).

**Figure 5:**
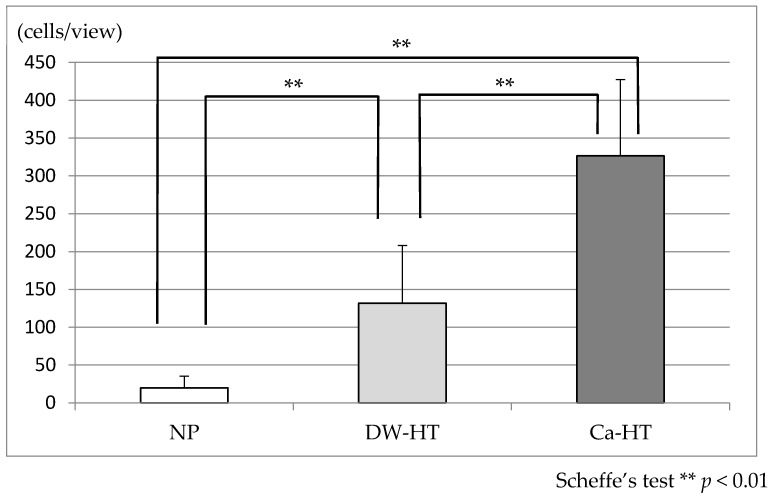
Adhesion of MC3T3-E1 osteoblastic cells onto the substrata after 1 h of incubation. The attached cell count for the Ca-HT group was significantly higher than for the NP and DW-HT groups. The attached cell count for the DW-HT group was significantly higher than for the NP group (*p* < 0.01).

After 1 h of incubation of MC3T3-E1 osteoblastic cell line with the substrata, the attached cell count for the Ca-HT group was significantly higher than for the other two groups. The attached cell count for the DW-HT group was significantly higher than for the NP group (Figure 5).

### 3.4. Cell Morphology

**Figure 6:**
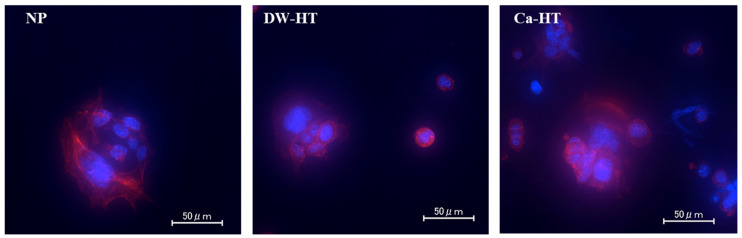
Fluorescent microscopic observation of GE1 cells seeded onto the substrata. After 12 h of incubation, cells were proliferating. There were no significant differences among the three groups.

After 12 h of incubation, GE1 cells were attached onto substrata and proliferating. There were no significant differences among the three groups (Figure 6).

**Figure 7:**
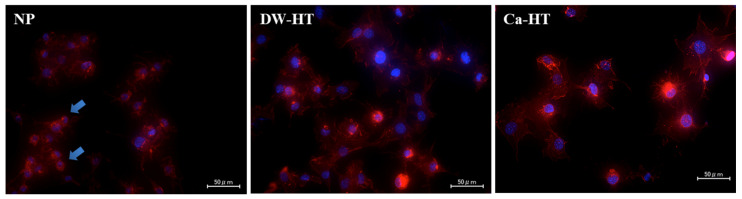
Fluorescent microscopic observation of MC3T3-E1 cells seeded onto the substrata. After 1 h of incubation, cells seeded onto both DW-HT and Ca-HT were well spread and their cell processes were prominent. Some cells seeded onto NP have also spread; however, others were still round in shape without extended cell processes (arrows).

After 1 h of incubation, MC3T3-E1 cells seeded onto DW-HT or Ca-HT plates predominantly exhibited spreading and expansion of cell processes. Some cells were also spread on the NP plates; however, others were still round-shaped, indicating incomplete cell adhesion onto the substrata (Figure 7).

### 3.5. Bacterial Adhesion

**Figure 8:**
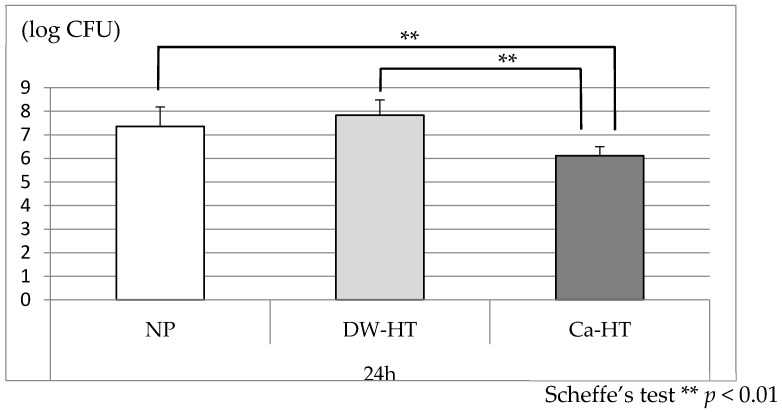
*Streptococcus gordonii* adhesion onto the substrata after 24 h of inoculation. The number of bacteria on Ca-HT was significantly lower than in the other two groups (*p* < 0.01). CFU: colony forming unit.

After 24 h of incubation, the bacterial count on the Ca-HT plates was significantly lower than those of the other two groups (Figure 8).

## 4. Discussion

ECM adsorption plays a crucial role in the adhesion of cells to the substratum [27]. In the present study, we hypothesized that hydrothermal treatment would provide a favorable surface for ECM protein adsorption onto a titanium substratum.

Laminin-332 is a major constituent of the basal lamina [28], which is interposed between the enamel and the junctional epithelium and plays a key role in epithelium–tooth adhesion. In vivo rodent studies revealed that laminin-332 was also observed between titanium implants and epithelial cells, but the expression was weak and, consequently, peri-implant epithelial bonding was much weaker than in the periodontium [8,9]. In the present study, the adsorption of rhLN onto the DW-HT surface was significantly higher than that for NP. According to the findings of a previous study, a denser laminin layer forms on hydrophilic (polyornithine-modified silicon dioxide) surfaces than on hydrophobic (alkanethiol-modified gold) surfaces [29]. Although these findings did not relate to titanium surfaces, a similar tendency was observed in our study.

In addition, rhLN adsorption was further enhanced on the Ca-HT titanium surface. Laminin-332 has been reported to have a Ca-binding site in its molecular structure [30], which may explain the higher level of rhLN adsorption onto Ca-HT titanium. On the contrary, the attached cell count of GE1 in the early stage was almost identical among the three groups. This is inconsistent with our previous in vitro study, in which we found that the epithelial cell attachment to Ca-HT titanium was more hardly detached than that to DW-HT [18]. Our in vivo rodent study also revealed that Ca-HT enhanced peri-implant soft tissue bonding [18]. Thus, this treatment may not promote epithelial cell attachment count, but strengthen cell attachment. Further research is required to elucidate this discrepancy.

The adsorption of rhOPN onto the DW-HT surface was also significantly higher than that for NP. Adsorption of rhOPN was further enhanced on the Ca-HT surface. Osteopontin, a major non-collagenous bone protein, is known to share cell adhesion activity with osteoblasts [31] and osteoclasts [32] via its arginine-glycine-asparaginic acid sequence. Osteopontin is further known to be involved in the development of the phenotype of osteoblastic cells and promote ossification [33]. In our previous in vivo study using immunoelectron microscopy, an amorphous layer approximately 70 nm thick was interposed between bone and the titanium, and osteopontin was one of the constituents of this layer [34]. Our results suggest that the hydrophilicity of both HT groups may play a role in rhOPN adsorption. In addition, our results showed that osteoblastic cell adhesion onto both HT surfaces was significantly enhanced. Studies have shown that surface hydrophilicity promotes osteoblastic cell adhesion [35,36,37] and differentiation [38], although the procedures to obtain surface wettability in these studies differed from ours. Our findings suggest that the enhanced adsorption of osteopontin onto the HT surface plays some role in osteoblastic cell adhesion.

In the present study, the adsorption of rhOPN onto the Ca-HT surface was further enhanced, and osteoblastic cell attachment also increased. Osteopontin has been reported to bind to hydroxyapatite [39] or calcium ions [40]. Thus, the surface calcium of Ca-HT titanium may contribute to the enhancement of rhOPN adsorption and subsequent cell attachment. This is consistent with a previous in vivo rodent study, in which Ca-HT titanium implants exhibited a higher bone–implant contact ratio [12].

BSA adsorption onto the substrata was higher for both HT surfaces than for NP titanium. A previous study also reported enhanced BSA adsorption onto titanium with an ultraviolet-generated super hydrophilic surface [35]. According to a previous study regarding the relationship between plasma proteins and biomaterials in the body, higher concentration proteins in the plasma were first adsorbed onto the biomaterial, and were subsequently replaced by other proteins with higher affinity for the biomaterial (the so-called “Vroman effect”) [41]. Furthermore, it was reported that protein adsorbed onto a hydrophilic substrata was easily replaced by other proteins; however, a protein adsorbed onto a hydrophobic surface underwent very little protein replacement [42]. To extrapolate our results according to the findings of previous reports, we hypothesized that BSA adsorbed onto the relatively hydrophobic NP surface would not be replaced readily by other proteins, but BSA on the hydrophilic HT surface would be replaced readily by other proteins with a high affinity for the substrata, such as osteopontin or laminin. Albumin possesses no cell adhesion capacity; therefore, if the replacement of adsorbed protein does not occur at the hydrophobic surface, cell adhesion onto the hydrophobic surface may be inhibited by the presence of albumin. In addition, a previous study also showed that albumin adsorbed onto a hydrophobic surface was easily removed by non-ionic detergent. A possible reason for this phenomenon is that albumin adsorbed onto the hydrophobic surface had its hydrophobic domain directed outward, allowing it to easily interact with non-ionic detergent [43]. This implies that, even after the adsorption of albumin onto the hydrophobic surface, the surface is still hydrophobic. It may be undesirable for osteoblastic or epithelial attachment because, according to several reports, these cells have a high affinity for hydrophilic substrata [44].

No difference in BSA adsorption between Ca-HT and DW-HT titanium surfaces was observed. This implies that the adsorption of BSA onto the substratum is independent of Ca. However, in comparison with DW-HT, Ca-HT can enhance the adsorption of both rhLN and rhOPN onto titanium without increasing BSA adsorption. This suggests that Ca-HT can improve both epithelial and osteoblastic cell adhesion onto titanium in an exclusive and promotive manner.

In the present study, a bacterial adhesion assay was performed using *Streptococcus gordonii. S. gordonii* was selected for this study because it is bacteria that rapidly adhere to the acquired pellicle formed on the tooth surface, and is followed by other bacteria that layer onto the initially-adhered bacteria to form the bacterial plaque [45]. In addition, *S. gordonii* is highly-detectable within the non-inflammatory peri-implant sulcus or within the implant components [46]. In the present study, the bacterial count on Ca-HT titanium was significantly lower than in the other two groups after 24 h of incubation. These findings contrast with the protein adsorption and cell adhesion experiments. The reason for this result remains unclear, but it can be speculated that the presence of calcium on the surface of the titanium influences the composition of the acquired pellicle from the saliva. These findings suggest that Ca-HT can enhance the biocompatibility of titanium as a dental implant, without enhancing bacterial adhesion. 

The major drawback of the present study was that we employed only one bacterial species. In the oral cavity, there are many conditions to be tested, such as the interaction between species and environmental variations such as anaerobic conditions. Multi species bacterial studies would be required to provide more evidence in regard. In addition, our procedure for the measurement of bacterial number could not distinguish live and dead bacteria. Further study is needed to clarify the cellular/bacterial events in the tissues surrounding the implant.

## 5. Conclusions

Ca-HT treatment could enhance osteoblastic cell adhesion. It can be speculated that the high affinity of Ca-HT titanium for rhOPN plays a role in this enhancement. Although Ca-HT was reported to strengthen epithelial cell attachment and in vivo peri-implant epithelial bonding, this treatment did not improve attached cell count of epithelial cells. High affinity of Ca-HT titanium for rhLN plays a role, not in attached cell count, but the strength of individual cell attachment. In spite of its enhancement of cell adhesion, Ca-HT titanium did not increase the adhesion of bacteria. This characteristic could be beneficial when Ca-HT is used to treat intraoral devices.
